# IgAim: cell surface Ig-aimed immune memory erasers for the therapy of autoimmune diseases and B leukemia

**DOI:** 10.1093/narmme/ugaf016

**Published:** 2025-04-30

**Authors:** Hiroshi Arakawa

**Affiliations:** IFOM ETS - The AIRC Institute of Molecular Oncology, 20139 Milan, Italy

## Abstract

Autoimmune diseases are caused by the misdirection of the immune system as the side effect of antibody diversity, wherein each B cell possesses unique characteristics defined by its antibodies. To address this issue, I have developed several cell surface immunoglobulin (Ig)-aimed immune memory erasers (IgAim) that consist of toxins explicitly targeted to cell surface Ig (sIg) on B cells. IgAim is created by replacing the receptor binding domain of diphtheria toxin with Staphylococcal protein A (SpA), affibodies, or specific antigens. The toxins IgAimG and IgAimA selectively killed sIgG^+^ and sIgA^+^ B cells, respectively. Treatment with these proteins kills between 90% and 99% of the target cell population without displaying toxicity toward other classes of B cells or nontarget cell types. The specificity of IgAim is highlighted by IgAimP7 which exclusively kills B cells expressing an antibody against pollen allergen Phleum pratense (Phl) p7. IgAim could be the basis of an effective future family of therapeutic agents for treating autoimmune diseases as well as B leukemia.

## Graphical Abstract

**Graphical Abstract:**
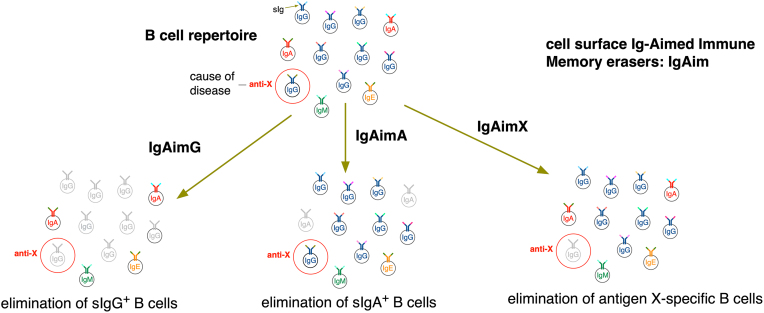

## Introduction

Antibodies possess remarkable diversity, enabling them to bind to a nearly limitless range of antigens. This variety of antibodies depends on complex mechanisms that edit the genome of individual B cells [1]. The variable regions of antibodies are generated by V(D)J recombination and further modified by somatic hypermutation, resulting in increased affinity following exposure to antigens. In addition to the diversity in the variable regions, there are five different classes of constant regions in human antibodies. The classes, excluding IgM and IgD, are the outcome of switch recombination. Alongside gene conversion [2], switch recombination and somatic mutation [3] are initiated by activation-induced cytidine deaminase (AID).

Of the antibody classes, IgM is the first to be expressed during B-cell development. Among them, the naive B cells have yet to encounter an antigen and represent the majority of the total B-cell repertoire [1]. However, in circulation in the blood, IgG is both the most abundant and has the longest serum half-life of all of the immunoglobulin (Ig) classes [1]. IgA plays a crucial role in protecting mucosal surfaces from viruses and bacteria [1]. The least prevalent class, IgE, deals with parasitic infections but is also associated with allergies and anaphylaxis [1].

The B-cell populations play a crucial role in acquired immunity, which primarily functions to combat foreign pathogens. However, B cells can also underly a number of pathologies, including allergy, autoimmune disease, and cancer [1]. Treatments for these B-cell maladies often involve targeting the entire B-cell population in a nonspecific manner [4]. This broad therapeutic approach can result in the elimination of large numbers of healthy, normal B-cell populations along with the pathological population. Notably, the pathogenic B cells implicated in these various diseases often originate from just a single B cell that expresses a unique Ig which can be considered a stable biological marker for that specific B-cell population. Thus, the development of an easily tunable therapeutic approach to specifically target a B cell expressing unique cell surface immunoglobulin (sIg) could provide a safe alternative to conventual therapies and allow for the selective elimination of pathogenic B-cell populations.

Canonically, B cells produce immunoglobulins that are then secreted and proceed to opsonize antigens they encounter. However, B cells can also employ endocytosis when exposed to an antigen. This can occur when a membrane-bound Ig on the B cell’s surface is exposed to a specific antigen. The antigen-antibody complex is then internalized and degraded. This process provides fragments of antigen that the B cell can then load on class II major histocompatibility complexes (MHCs) and present to helper T cells [1]. This propensity for B cells to employ antibody-mediated endocytosis of specific antigens, along with their clone-specific, unique, and homogeneous cell surface immunoglobulins, may provide a valuable avenue for therapeutic intervention.

While membrane-bound and secreted immunoglobulins are produced by alternative splicing of the same Ig genes, sIg^+^ B cells express membrane-bound antibodies on their cell surface as receptors to recognize specific antigens. This study aims to identify a way to target sIg^+^ B cells specifically and, once identified, eliminate the target cell population specifically. To accomplish this, I employed a conjugated subunit of the diphtheria toxin. Diphtheria toxin is naturally produced by *Corynebacterium diphtheria*. The A fragment (N-terminal) of diphtheria toxin, DtA, contains a catalytic domain that disrupts protein synthesis in eukaryotic cells resulting in cytotoxicity in susceptible cells [4]. The B fragment of the diphtheria toxin (C-terminus) consists of a transmembrane domain and a receptor-binding domain that facilitates the translocation of the catalytic domain from the endosome to the cytoplasm. As diphtheria toxin is not toxic to mice that naturally do not have its receptor, its toxicity requires binding to the specific receptor [5]. By replacing the receptor-binding domain of diphtheria toxin with cytokines, growth factors, cancer-specific cell-penetrating peptides, and cancer cell antigens, this toxin has already been exploited as a therapeutic agent [4].

Staphylococcal protein A (SpA) [6] and Streptococcal protein G (SpG) [7] are commonly used molecules for binding antibodies, for example, in immunoprecipitation, enzyme-linked immunosorbent assay (ELISA), and western blotting. SpA binds to immune complexes and serum immunoglobulins, making it widely utilized in biochemical, biotechnological, and medical applications [6]. Additionally, SpA can directly bind to antibodies on B cells [6]. It has been reported that diphtheria toxin fused with the SpA ZZ domain [8] or truncated Pseudomonas exotoxin A linked to the SpA ZZ domain [9] exhibit mild toxicity to cells through binding to antibodies against cell surface antigens. However, their toxicity to B cells remains unknown.

In this study, I developed a strategy to leverage unique features of B-cell biology and the cytotoxic effect of diphtheria toxin to target specific populations of B cells. I call this new class of drugs Ig-aimed immune memory eraser(s) (IgAim). I have demonstrated *in vitro* the specificity and efficacy of IgAim to recognize and eliminate B-cell populations based on antigen specificity and Ig classes.

## Materials and methods

### Design and construction of IgAim

The control vector was constructed by introducing amino acid substitutions of E82K and K179E into pET-22b DT 51E/148K (Addgene, #11081) to revert the inactive diphtheria toxin to the active form and by deleting amino acids 2–31 and 418–566, corresponding to the pel B signal peptide and the receptor binding domain, respectively. The XhoI site between the translocation domain and the His-tag of the resulting protein was used for cloning fusion partner proteins. The following proteins were used as fusion partners: SpA; SpA ZZ domain of pKK-TEV-ProteinA [10] (addgene #105788), SpG, amino acids 17–73 of in pET His6 ProteinG TEV LIC cloning vector (1P) (Addgene, #29660), ZigA; mutations according to Gunneriusson *et al.* [11] were introduced in SpA sequence, Phl p7 full-length coding sequence of 78 amino acids (Y17835). The anti-Phl p7 human monoclonal antibody expression vector was constructed by antibody light and heavy chain polymerase chain reaction (PCR) product into a BOS-promoter lentiviral expression vector, where the antibody light chain gene and heavy chain VDJ gene are derived from pVITRO1-102.1F10-IgE/lambda (Addgene #50365), membrane-type Cgamma1 gene from KARPAS-422 cDNA, and a furin T2A linker from oligos. The GGGS linker (SSGGGSSGGGS), the furin T2A linker (RRKRGSGLINEGRGSLLTCGDVEENPGP), and the Phl p7 gene were created by PCR amplification of overlapping oligos. pET-22b DT 51E/148K was a gift from John Collier (Addgene plasmid #11081; http://n2t.net/addgene:11081; RRID:Addgene_11081). pKK-TEV-ProteinA was a gift from Andrzej Dziembowski (Addgene plasmid #105788; http://n2t.net/addgene:105788; RRID:Addgene_105788). pET His6 ProteinG TEV LIC cloning vector (1P) was a gift from Scott Gradia (Addgene plasmid #29660; http://n2t.net/addgene:29660; RRID:Addgene_29660). pVITRO1-102.1F10-IgE/λ was a gift from Andrew Beavil (Addgene plasmid #50365; http://n2t.net/addgene:50365; RRID:Addgene_50 365).

### Protein purification

IgAim expression vectors were transformed into T7 Express Competent *Escherichiacoli* (High Efficiency) (NEB, C2566H). Logarithmically growing bacteria were incubated with 0.4 mM Isopropyl β-D-1-thiogalactopyranoside (IPTG) for 2 h at 37°C to induce IgAim protein expression. *E. coli* were lysed by NEBExpress *E. coli* Lysis Reagent (NEB, P8116S), and His-tagged proteins were purified from the soluble fraction by NEBExpress Ni Spin Columns (NEB, S1427L) or NEBExpress Ni Resin (NEB, S1428S). Protein concentrations were measured with Qubit Protein Assay Kit (Thermo Fischer, Q33211). The purity of the proteins of appropriate sizes was quantified using Image Lab Software (Bio-Rad).

### Culture of the cell lines

The culture medium for B and other suspension cell lines is RPMI with 10% fetal bovine serum, 1% L-glutamine, 1% penicillin/streptomycin, and 0.05 mM 2-mercaptoethanol. The culture medium for adherent cell lines is DMEM with 10% fetal bovine serum, 1% L-glutamine, and 1% penicillin/streptomycin. Cell lines were cultured at 37°C with 5% CO_2_. Packaging and transduction of the anti-Phl p7 monoclonal antibody expression lentiviral vector were performed according to the previously described [12]. The vector was transfected into the sIg^−^ B-cell line, Raji, and sorted the sIg^−^ population of the Ramos B-cell line, and the clones highly expressing the Ig were selected by limiting dilution.

### Peripheral blood mononuclear cell culture

Primary peripheral blood mononuclear cells (PBMCs) [ATCC (LGC Standards S.r.L.), PCS-800–011] were washed once with RPMI medium containing 20% FBS and then cultured at 37°C for 1 h in a Falcon tube in 10 ml of RPMI medium containing 150 U of DNase I to degrade DNA released from dead cells that can cause cell clumps. After washing again with the RPMI medium with 10% fetal bovine serum, 1% L-glutamine, 1% penicillin/streptomycin, and 0.05 mM 2-mercaptoethanol, the cells were cultured in the RPMI medium.

### Cell viability assay

Cells at a concentration of 50 000 cells/ml were cultured in triplicate in 96-well microtiter plates with different concentrations of IgAim. Human Serum (Merck, S1-100ML) and IgG from human serum (Sigma, I4506-10MG) were used to analyze the effect of human serum or IgG. After ∼70 h of culture with IgAim, 50 μl of suspension cells or 100 μl of adherent cells were used for viability assays. For these volumes of cells, I initially used 50 μl of CellTiter-Glo Luminescent Cell Viability Assay reagent (Promega, G7571). Still, during this project, I found that 20 μl of the CellTiter-Glo reagent provided sufficient quantitation, so some experiments were performed using the 20 μl of reagent instead of 50 μl. Cells were incubated with the CellTiter-Glo reagent for 20 min at room temperature, and luminescence was measured on a Victor 3 plate reader (PerkinElmer).

### Selective elimination of B cells from mixed cells

Ramos, U-2904, and Raji anti-P7 were mixed at a ratio of 1:2:2 (total 50 000 cells/ml) considering the proliferation rates of each cell type and cultured in a six-well plate in the presence of 1 μg/ml IgAimG, IgAimA or IgAimP7 or in the absence of IgAim. PBMCs were cultured in a 12-well plate at a concentration of 10^6^ cells/ml in the presence of 1 μg/ml IgAimG or IgAimA or the absence of IgAim. The survival ratios of these cells were measured by the flow cytometry.

### Enzyme-linked immunosorbent assay (ELISA)

ELISA was performed using the Human IgG ELISA Kit 24T (Elabscience, E-EL-H0169) and the QuicKey Pro Human IgA ELISA Kit 24T (Elabscience, E-OSEL-H0009) according to the manual provided by the manufacturer.

### Flow cytometry

The following antibodies and SpA were used to test the antibody class of B cells: Goat anti-Human IgG (Fc specific)-Cy3 antibody (Merck, C2571-1ML), Goat anti-Human IgG (Fc specific)-PerCP antibody (Jackson ImmunoResearch, 109-126-098), Goat anti-Human IgM (mu-chain specific)-FITC antibody (sigma, F5384-1ML), Goat anti-Human IgA (alpha-chain specific)-FITC antibody (Sigma, F5259-1ML), Goat anti-Human IgE Fc secondary antibody (FITC) (Invitrogen, #H15701), Mouse anti-Human CDC19-APC monoclonal antibody (Merck, SAB4700110-100TST), and Protein A–FITC conjugate (Thermo Fisher, 101011). Flow cytometry was performed on a FACS Calibur (Becton Dickinson).

Among the two different anti-IgG antibodies described above, the PerCP antibody was used for IgG analysis of PBMCs because the PerCP anti-human IgG antibody had a lower nonspecific background than the Cy3 antibody.

The flow cytometry was initially performed using the FACS Calibur (Becton Dickinson) for Ig class identification of B-cell lines; however, because during the research period, the equipment broke down and became unusable, the Attune NxT Flow Cytometer (Thermo Fisher Scientific) was subsequently used for analysis of mixed B-cell lines and PBMCs.

## Results

### IgAim: cell surface Ig-aimed immune memory erasers

**Figure 1.**
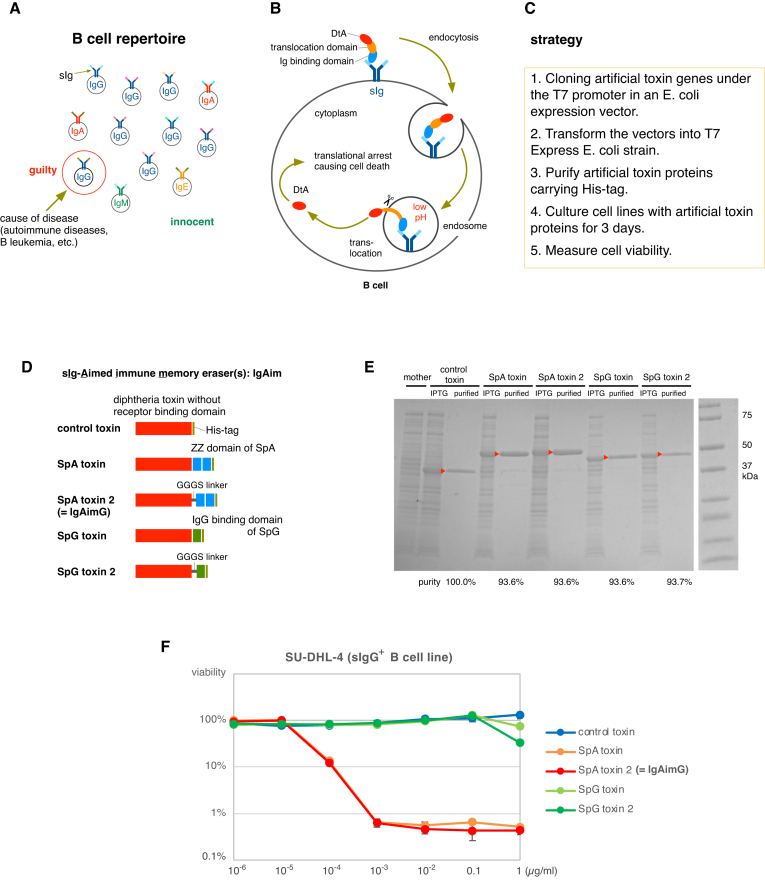
IgAim: cell surface Ig-aimed immune memory erasers. (**A**) Illustration depicting the uniqueness of B cells. Cell surface Ig (sIg) is a membrane-bound Ig, and its antibody class and antigen specificity can distinguish each sIg^+^ B cell. (**B**) Expected mechanism of action of the antibody-binding toxin. After binding to the antibody, the toxin is internalized into the cell by endocytosis and translocated into the cytoplasm, causing translation arrest and cell death. (**C**) Overview of the strategy. (**D**) Design of IgAim. One control toxin and four different toxins against sIgG^+^ B cells. The SpA toxin 2 was renamed to IgAimG. (**E**) IPTG induction and purification of IgAim proteins. Purified toxin proteins are highlighted by triangles. (**F**) Evaluation of IgAim cytotoxicity against the sIgG^+^ B-cell line SU-DHL-4. Cells were cultured for 3 days with IgAim, and the viability was measured by the luminescent cell viability assay. Experiments were performed in triplicate.

Each B cell can be distinguished by the antigen specificity or the class of its antibodies (Fig. 1A). Figure 1B shows the expected mechanism of action of antibody-binding toxins derived from diphtheria toxin. After binding to the antibody, the artificial toxin is translocated to endosomes by receptor-mediated endocytosis. The acidic pH of the endosome causes a conformational change in the transition domain and membrane, allowing the release of DtA into the cytoplasm [4]. The strategy for erasing immune memory by selectively removing B cells is summarized in Fig. 1C. Diphtheria toxin fusion genes are cloned into an E. coli expression vector under the control of the T7 promoter, followed by transformation into T7 Express *E. coli*. After the fusion protein expression was induced by IPTG, these toxins containing a His tag were purified using Ni-NTA spin columns. Cell lines were cultured in the presence of the engineered toxin proteins for 3 days, and cell viability was measured (Fig. 1C).

SpA exhibits binding affinity to all IgG subclasses, except for IgG3 [6], whereas SpG can bind to all subclasses of human IgG antibodies [7]. In this study, one control toxin and four toxins to target sIgG^+^ B cells were designed (Fig. 1D). A GGGS linker was inserted between the diphtheria toxin catalytic domain and SpA/SpG in toxins SpA toxin 2 and SpG toxin 2. These artificial toxins were identified as additional bands after IPTG induction and were purified using Ni-NTA spin columns (Fig. 1E).

The cytotoxicity of these proteins, named Ig-aimed immune memory eraser(s), IgAim, was assessed in the sIgG^+^ B-cell line SU-DHL-4 (Fig. 1F). Cells were cultured for 3 days with IgAim, and viability was measured by luminescent cell viability assay, which quantifies the number of viable cells based on their ATP levels. Control toxins did not exhibit any toxicity. The mechanism of diphtheria toxin cytotoxicity involves translocating the toxic domain to the cytoplasm through a conformational change in the translocation domain at acidic pH. If the toxin domain is nonspecifically taken up by cells via endocytosis, it may remain in the endosomes without resulting in toxicity [4]. The SpG-toxin showed weak cytotoxicity at a 1 μg/ml concentration. In contrast, the SpA-toxin showed significant toxicity at concentrations as low as 0.0001 μg/ml and effectively killed 99% of cells >0.001 μg/ml. Consequently, the SpA-toxin was renamed IgAimG and investigated further to evaluate its toxicity against other sIgG^+^ B-cell lines.

### IgAimG: sIgG^+^ B-cell eraser

Antibody classes and SpA binding were evaluated in a panel of 30 B-cell lines (Supplementary Fig. S1), identifying 12 of sIgG^+^ B-cell lines. Subclasses of sIgG^+^ B-cell lines were further identified by partial sequencing of their Cγ complementary DNA (cDNA). It has previously been reported that the serum levels of four IgG subclasses, IgG1, IgG2, IgG3, and IgG4, are ∼67%, 22%, 7%, and 4% of total IgG levels, respectively [1]. While IgG4 is typically the least abundant subclass in physiological conditions, it is noteworthy that several IgG4-expressing B-cell lines were present, suggesting a possible pathogenic relationship between B lymphoma with chronic inflammation due to IgG4-related diseases [13].

**Figure 2.**
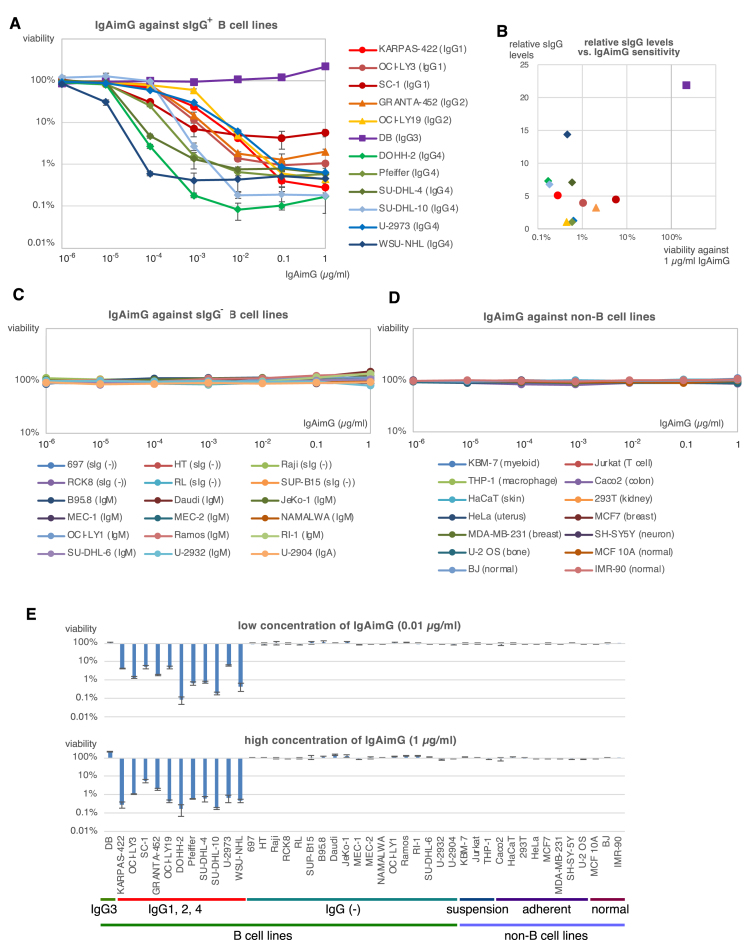
IgAimG: sIgG^+^ B-cell eraser. (**A**) The cytotoxicity of IgAimG to the sIgG^+^ B-cell lines. The IgG subclasses of the B-cell lines are also indicated. Circles, triangles, squares, and diamonds represent the B-cell subclasses, IgG1, IgG2, IgG3, and IgG4, respectively. (**B**) IgG expression levels of sIgG^+^ B cells were evaluated using the flow cytometry and normalized. These sIgG levels of cell lines are plotted against their viability in the presence of 1 μg/ml IgAimG in Fig. 2A. The symbols for each cell line are the same as in Fig. 2A. (**C**) The cytotoxicity of IgAimG to the sIgG^−^ B-cell lines. The Ig classes of the B-cell lines are also indicated. (**D**) The cytotoxicity of IgAimG to non-B cell lines. The origin or other characteristics of the cell lines are also indicated. (**E**) Comparison of the cytotoxicity of high and low concentrations of IgAimG to B-cell lines and other cell lines. In Fig. 2A, C, and D, cells were cultured for 3 days with IgAimG, and the viability was measured by the luminescent cell viability assay. These experiments were performed in triplicate, although error bars were not shown in Fig. 2C and D.

The sIgG^+^ B-cell lines were subjected to a three-day culture in the presence of IgAimG. IgAimG showed potent cytotoxicity to all the sIgG^+^ B-cell lines except for the IgG3 B-cell line DB (Fig. 2A). The resistance of DB to IgAimG is likely due to the inability of SpA to bind IgG3 [6]. Indeed, despite its high levels of sIgG, DB did not bind to SpA (Supplementary Fig. S1). Figure 2B plots the sIgG levels of each sIgG^+^ B-cell line evaluated by the flow cytometry against IgAimG sensitivity, revealing a partial correlation between sIgG expression levels and IgAimG sensitivity. IgG4 B cells (diamonds in Fig. 2B) were relatively more sensitive to IgAimG. Interestingly, cells with low levels of sIgG expression (U-2973, Pfeiffer, OCI-LY19) were also susceptible to killing by IgAimG (Fig. 2B). It is plausible that not only the level of sIgG expression but also the efficiency of sIgG endocytosis plays a role in its efficacy [14].

IgAimG did not show toxicity to sIgG^−^ B-cell lines, namely sIg^−^, sIgM^+^, and sIgA^+^ B-cell lines (Fig. 2C). Furthermore, IgAimG did not show toxicity toward non-B cell lines derived from various organs (Fig. 2D). Figure 2E summarizes the results of Fig. 2A, C, and D; IgAimG did not exert toxicity on non-IgG-expressing cells even at high concentrations (1 μg/ml), whereas it efficiently killed sIgG^+^ B cells at low concentrations (0.01 μg/ml). Additionally, IgAimG combined with the supernatant from sIgG^+^ B cells did not exhibit nonspecific toxicity, indicating that IgAimG killings are mediated by cell surface IgG rather than cell-free IgG (Supplementary Fig. S2A). Cell contents released from IgAimG-killed sIgG^+^ B cells were not toxic to nonspecific cells (Supplementary Fig. S2B). Thus, the toxicity of IgAimG appears to be specific to sIgG^+^ B cells of IgG1, G2, and G4 subclasses.

### IgAimA: sIgA^+^ B-cell eraser

Replacement of 13 surface residues of SpA can change its binding specificity to different target proteins [15]. Proteins made by randomization of 13 amino acids of SpA and screened by altered binding specificities are called affibodies [15]. Thus, affibodies, similar to antibodies, are engineered to possess diverse binding specificities. Among these affibodies, ZigA is specifically designed to bind exclusively to IgA and not IgG [11].

**Figure 3.**
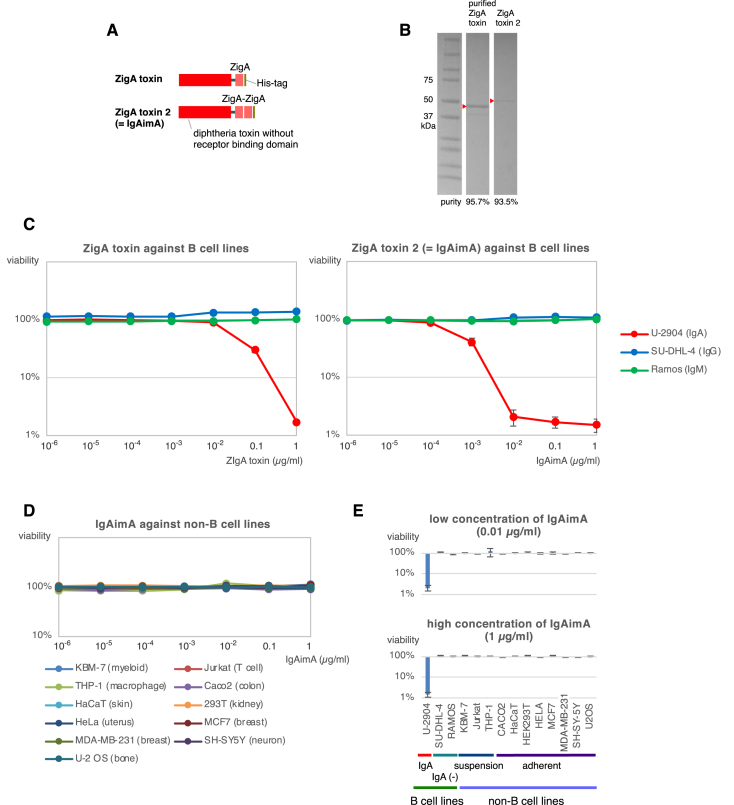
IgAimA: sIgA^+^ B-cell eraser. (**A**) Two toxins using affibodies against IgA (ZigA). The ZigA toxin 2 was renamed to IgAimA. (**B**) Purification of the toxin proteins. Purified toxin proteins are highlighted by triangles. (**C**) The cytotoxicity of IgA toxins against the B-cell lines. (**D**) The cytotoxicity of IgAimA to non-B-cell lines. (**E**) Comparison of the cytotoxicity of high and low concentrations of IgAimA to B-cell lines and other cell lines. In Fig. 3C and D, cells were cultured for 3 days with IgAim, and the viability was measured by the luminescent cell viability assay. These experiments were performed in triplicate, although error bars were not shown in Fig. 3D.

I substituted the receptor binding domain of the diphtheria toxin with a monomer or a tandem repeat of ZigA, which can specifically bind human Cα (Fig. 3A). These artificial toxin proteins were purified, as shown in Fig. 3B. ZigA toxin showed toxicity to a sIgA^+^ B-cell line, U-2904, at a concentration of 0.1 μg/ml and killed nearly 99% of the cells at 1 μg/ml (Fig. 3C, left). The toxic activity was further enhanced by the tandem ZigA in ZigA toxin 2, which displayed toxicity at a concentration of 0.001 μg/ml, effectively killing nearly 99% of IgA^+^ B cells at a concentration of 0.01 μg/ml (Fig. 3C, right). The ZigA toxin 2 was therefore renamed to IgAimA. Notably, the toxicity of IgAimA was specific to IgA^+^ B-cell line. Other B-cell classes and non-B-cell lines were unaffected by IgAimA (Fig. 3C–E).

### IgAimP7: anti-allergen B-cell eraser

**Figure 4.**
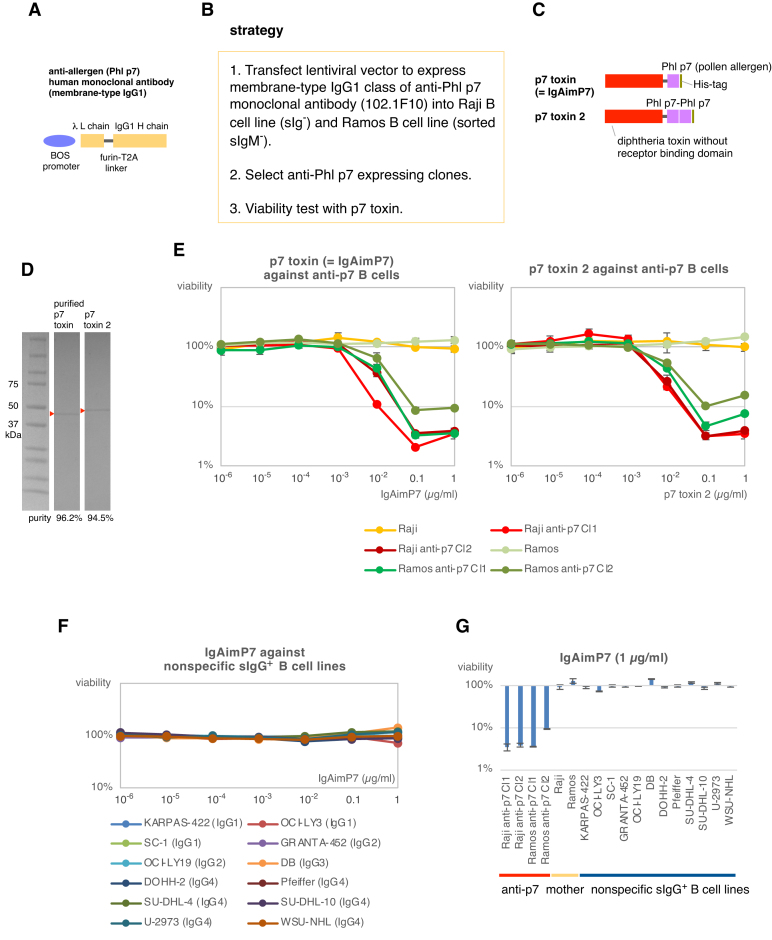
IgAimP7: anti-allergen B-cell eraser. (**A**) A lentiviral vector to express human monoclonal antibody against the pollen allergen Phl p7. (**B**) Overview of the strategy. The anti-Phl p7 monoclonal antibody expression vector was transfected into the sIg^−^ B-cell line, Raji, and the sorted sIgM^−^ population of the Ramos B-cell line. (**C**) Design of two toxins using Phl p7. The p7 toxin was renamed to IgAimP7. (**D**) Purification of the toxin proteins. Purified toxin proteins are highlighted by triangles. (**E**) The cytotoxicity of p7 toxins against the B-cell lines overexpressing anti-p7 IgG1 antibody. (**F**) The cytotoxicity of IgAimP7 to nonspecific sIgG^+^ B-cell lines. (**G**) Comparison of the cytotoxicity of IgAimP7 to anti-p7 specific and nonspecific sIgG^+^ B-cell lines. In Fig. 4E and F, cells were cultured for 3 days with IgAim, and viability was measured by luminescent cell viability assay. These experiments were performed in triplicate, although error bars were not shown in Fig. 4F.

To selectively eliminate antigen-specific B cells, the receptor binding domain of diphtheria toxin was replaced by an antigen. A human antibody gene [16] specific for Phl p7 [17], a common allergen causing pollinosis, was cloned into a lentiviral vector and expressed under the BOS promoter, as illustrated in Fig. 4A. The vector was designed to enable post-translational separation of the Ig light and heavy chains through a linker containing two cleavage sites of furin and T2A [18]. The antibody expression vector was subsequently introduced into sIg^−^ Raji B cells or sorted sIgM^−^ Ramos B cells (Fig. 4B). Single and tandem variants of the Phl p7-toxin were designed (Fig. 4C), and proteins purified (Fig. 4D). Both p7 toxin and p7 toxin 2 displayed toxicity against anti-p7 Raji and anti-p7 Ramos cells at concentrations of 0.01 μg/ml, effectively killing over 90% of anti-p7 B-cell lines at 0.1 μg/ml (Fig. 4E). The cytotoxic effects were similar for both p7 toxin variants; the toxin containing single Phl P7 (IgAimP7) is sufficient to kill anti-p7 B cells (Fig. 4E, left). Importantly, they did not exhibit toxicity toward their mother cell lines or nonspecific sIgG^+^ B-cell lines (Fig. 4E–G).

### Effect of human serum on the cytotoxicity of IgAim

**Figure 5.**
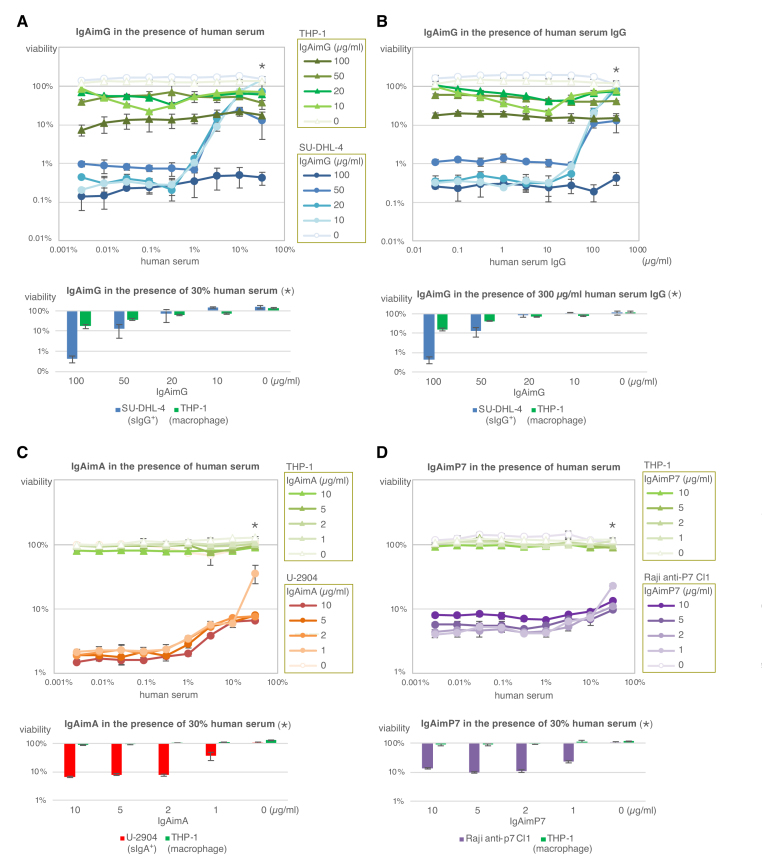
Effect of human serum and IgG on the cytotoxicity of IgAim. (**A**) The cytotoxicity of IgAimG to the sIgG^+^ B-cell line SU-DHL-4 and the macrophage cell line THP-1 in the presence of human serum. (**B**) The cytotoxicity of IgAimG to SU-DHL-4 and THP-1 in the presence of human serum IgG. The symbols are the same as in Fig. 5A. (**C**) The cytotoxicity of IgAimA to the sIgA^+^ B-cell line U-2904 and macrophage cell line THP-1 in the presence of human serum. (**D**) The cytotoxicity of IgAimP7 to anti-p7 Raji Cl1 and macrophage cell line THP-1 in the presence of human serum. Cells were cultured with IgAimG, IgAimA, or IgAimP7 in the presence of human serum or human serum IgG for 3 days, and viability was measured by luminescent cell viability assay. The data marked with asterisks in the upper graphs (A–D) are summarized in the graphs below.

To test the effect of human serum on the cytotoxicity of IgAimG, the sIgG^+^ B-cell line SU-DHL-4 and macrophage cell line THP-1 were cultured with IgAimG in the presence of human serum, and cell viabilities were measured after 3 days (Fig. 5A). Viabilities in 30% human serum (*) are extracted from the upper graph and summarized below (Fig. 5A). Higher concentrations of IgAimG were required to kill SU-DHL-4 in the presence of human serum; 50 μg/ml IgAimG killed nearly 90% of SU-DHL-4 in the presence of high concentrations of human serum, and 100 μg/ml IgAimG killed >99% of the SU-DHL-4 (Fig. 5A). However, these concentrations of IgAimG were mildly toxic to THP-1 (Fig. 5A). Because of unknown reasons, IgAimG sometimes showed mild toxicity to THP-1 at relatively low serum concentrations, but this toxicity disappeared with higher serum concentrations (Fig. 5A, upper).

IgAimG was also affected by human IgG (Fig. 5B), similar to human serum (Fig. 5A). The concentrations of IgG and IgA in the human serum measured by ELISA were 17.0 and 1.05 mg/ml, respectively. Thus, because human serum contains high concentrations of IgG, most probably human IgG interferes with the action of IgAimG, as IgAimG is designed as a toxin that binds to the Fc region of IgG. Nevertheless, 50 μg/ml IgAimG killed nearly 90% of sIgG^+^ B cells even in high concentrations of human IgG, and 100 μg/ml IgAimG killed >99% of SU-DHL-4 (Fig. 5B).

IgAimG at 10 or 20 μg/ml was not toxic to SU-DHL-4 in the presence of high concentrations of human serum (Fig. 5A) or human IgG (Fig. 5B). Notably, no toxicity to macrophage cell lines was observed at these concentrations of IgAimG (Fig. 5A and B), suggesting that the IgG/IgAimG complex is not cytotoxic to macrophages. In addition to killing sIgG^+^ B cells, IgAimG may inactivate human IgG because SpA is an immunosuppressive protein that blocks IgG.

The human serum did not affect strongly on the cytotoxicity of IgAimA, probably due to the limited concentration of IgA in the serum: even 1 μg/ml IgAimA killed ∼60% of sIgA^+^ B-cell line, U-2904, and IgAimA above 2 μg/ml killed over 90% of U-2904 (Fig. 5C). IgAimA up to 10 μg/ml was not toxic to THP-1 in the presence of human serum (Fig. 5C). A similar trend was seen with IgAimP7: even 1 μg/ml IgAimP7 killed ∼80% of the Raji B-cell line expressing anti-P7 antibody, and IgAimP7 above 2 μg/ml killed over 90% of this cell line (Fig. 5D). Up to 10 μg/ml IgAimP7 was not toxic to THP-1 in the presence of human serum (Fig. 5D).

### Selective elimination of B cells from mixed B-cell lines of different classes

**Figure 6.**
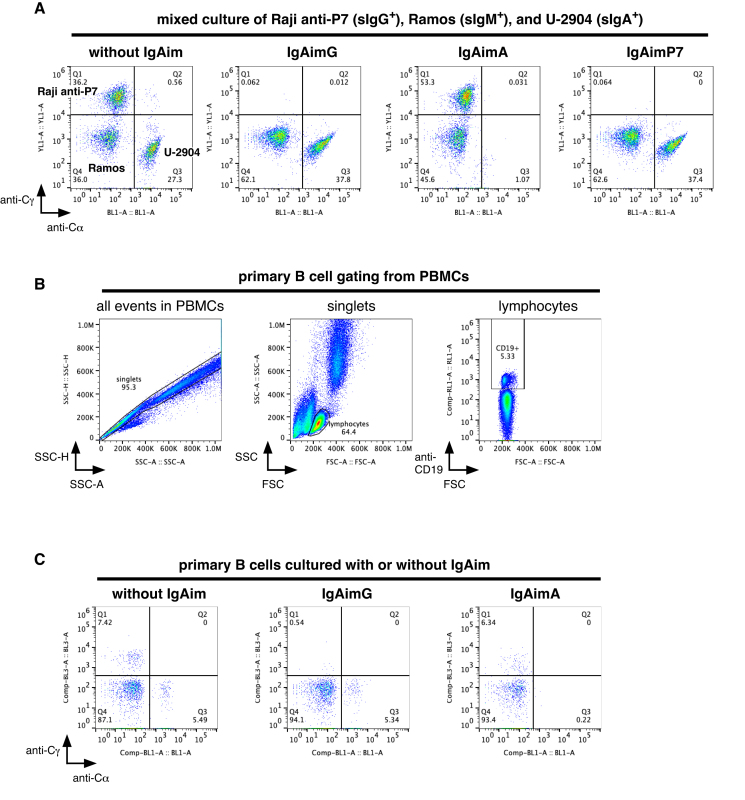
Selective removal of B cells from mixed cells using IgAim. (**A**) Three different classes of B-cell lines, namely sIgM^+^ B-cell line Ramos, sIgA^+^ B-cell line U-2904, and sIgG^+^ B-cell line anti-P7 Raji, were mixed in a 1:2:2 ratio considering the proliferation rates of each cell type. These mixed cell lines were cultured for 3 days in the presence of 1 μg/ml IgAimG, IgAimA, and IgAimP7. The proportions of each cell line were analyzed by the flow cytometry. (**B**) Lymphocytes were identified from PBMCs based on morphological parameters, and primary B cells were then gated based on CD19 expression. (**C**) PBMCs were cultured for 3 days in the presence of 1 μg/ml IgAimG and IgAimA, and the proportions of sIgG^+^ and sIgA^+^ primary B cells were analyzed by the flow cytometry.

To investigate whether IgAim can selectively eliminate specific B cells, three different classes of B-cell lines were co-cultured: the sIgG^+^ B-cell line anti-P7 Raji, sIgM^+^ B-cell line Ramos, and sIgA^+^ B-cell line U-2904. Since Ramos proliferates faster than the other cell lines, Ramos, anti-P7 Raji, and U-2904 were mixed in a 1:2:2 ratio. Each cell line was identified by staining with anti-Cγ and anti-Cα antibodies, resulting in three distinct clusters (Fig. 6A). These mixed cell lines were cultured for three days with 1 μg/ml IgAimG, IgAimA, or IgAimP7 or without IgAim, and the proportions of each cell line were analyzed by the flow cytometry. IgAimG selectively eliminated the sIgG^+^ B-cell line anti-P7 Raji while retaining Ramos and U-2904 (Fig. 6A). Similarly, IgAimA selectively eliminated sIgA^+^ B-cell line U-2904, with no apparent damage or reduction in other cells. Anti-P7 Raji was also able to be selectively eliminated by P7 antigen-specific IgAimP7.

### Selective elimination of primary B cells in peripheral blood mononuclear cells (PBMCs)

To determine whether IgAim can selectively eliminate primary B cells belonging to a specific class, PBMCs were cultured for 3 days with 1 μg/ml IgAimG or IgAimA or without IgAim. PBMCs cultured without IgAim were first gated to singlets, then gated into the lymphocytes based on morphological parameters, and finally gated into CD19^+^ cells to identify primary B cells (Fig. 6B).

IgAimG selectively eliminated sIgG^+^ B cells from primary B cells, reducing their proportion from 7.42% to 0.54% while retaining sIgA^+^ cells and sIgG^−^ B cells (Fig. 6C). Similarly, IgAimA selectively eliminated sIgA^+^ B cells from primary B cells, reducing the proportion of sIgA^+^ B cells from 5.49% to 0.22% while retaining sIgG^+^ cells and sIgA^−^ B cells. IgAim was thus able to eliminate primary B cells selectively.

## Discussion

In this work, I developed a class of artificial toxins, termed IgAim, that can be customized and specifically targeted to the antibodies on the surface of B cells. IgAim can be tailored to eliminate monoclonal B-cell populations by targeting the antigen-binding site, or IgAim can be employed more broadly to eliminate entire classes of B cells.

Here, the term “immune memory” does not refer to canonical memory B cells, and the purpose of IgAim is not to remove all the immune memories. The memories in the brains of people suffering from trauma are not only bad memories; other memories are also important. IgAim aims to selectively remove “bad memories” from the B-cell repertoire.

The IgAim control toxin was not toxic to the sIgG^+^ B-cell line (Fig. 1F). IgAimG was toxic only to sIgG^+^ B cells of the IgG1, IgG2, and IgG4 subclasses (Fig. 2A), and IgAimA was toxic only to sIgA^+^ B cells (Fig. 3C). Importantly, none of these IgAims were toxic to other B cells or non-B cells (Figs 2C–E, 3C–E, and 6). IgAimP7 was toxic to B cells expressing anti-Phl p7 antibodies but not to their parental population or nonspecific sIgG^+^ B-cell lines (Figs 4E–G and 6A). Thus, the toxicity of IgAim depends on binding to the cell surface antibodies.

The low killing activity of SpG toxin and SpG toxin 2 against sIgG^+^ B cells (Fig. 1F) may be partly due to the presence of bovine IgG in the FBS because SpG binds much more strongly to bovine IgG than SpA. Additionally, because these SpG toxins contain only one IgG-binding domain, adding this domain might enhance its function.

Supernatants from sIgG^+^ cells killed by IgAimG showed no nonspecific toxicity (Supplementary Fig. S2), indicating that the IgAim-killed cells do not spread cytotoxic components around. Stochastic uptake of IgAim via random endocytosis appears insufficient to induce cytotoxicity. Since diphtheria toxin does not harm mice, which inherently lack diphtheria toxin receptors, IgAim is considered to have low nonspecific toxicity *in vivo* [5]. IgAimG does not show substantial toxicity to macrophages, even with human serum or IgG (Fig. 5A and B), indicating that IgAimG–IgG complexes, which may not bind the Fc receptor or be degraded in the phagosome, are not toxic to macrophages.

The ideal delivery target for *in vivo* administration of IgAimG is the B cell-rich organ, such as the spleen. Alternatively, removing antibodies from the serum by plasma exchange before administration may be helpful. In addition, repeated administration of IgAimG may work to block free antibodies and kill B cells, erasing broad immune memories. In contrast, the serum's effect on IgAimA or IgAimP7 was limited (Fig. 5C and D). Since each specific antibody is a small fraction of the total antibody, another helpful solution is to focus on antigen-specific B cells.

Several potential precautions are necessary for actual therapeutic use *in vivo*. Because of the formation of complexes with IgAim and antibodies in blood or tissue fluid, continued administration of the IgAim might ultimately lead to the onset of immune complex disease. If the IgAim binds simultaneously to target molecules/cells, cytokine storm syndrome might occur. These factors must be considered when determining the administration method and dosage of IgAim. IgA is mainly located in mucosa-associated lymphoid tissue (MALT), so IgAimA cannot target those cells unless it reaches MALT. In other words, it is possible to preserve IgA-positive cells within MALT while primarily targeting IgA-positive cells circulating in the blood. While repeated administration of IgAim may induce antibodies against the diphtheria toxin domain of IgAim, which may inhibit the action of IgAim, this reaction might be compensated by the destruction of B cells that produce these antibodies. To understand the safety and efficacy of the IgAim strategy under more physiological conditions, further *in vivo* studies are necessary.

IgAim has the ability to eliminate antigen-specific memory selectively. Notably, the use of IgAimG or IgAimA does not harm IgM class of B cells (Figs 2C and 3C), thereby preserving the naive B-cell repertoire, which constitutes the majority of the B-cell repertoire. The selective elimination of B cells holds promise for treating immune disorders without inducing general immunodeficiency. This approach has potential applications in various therapeutic contexts, for example, IgA nephropathy caused by antibodies of the IgA class.

One application of IgAim is to eliminate extensive immune memory. Removing sIgG^+^ B cells can effectively erase the overall adverse immune “trauma” associated with B cells. It is important to note that IgAimG does not kill IgG3^+^ B cells. However, IgG1, IgG2, and IgG4 collectively constitute ∼93% of the total IgG in the bloodstream [1]. Targeting these three IgG subclasses could significantly mitigate the harmful immune response by sIgG^+^ B cells.

This study’s B-cell lines killed by IgAimG are derived from B leukemia and lymphoma (Figs 2A and 3C). Numerous B leukemia cells depend on signaling from these cell surface antibodies and express them on their cell surface [19]. B leukemias expressing IgG or IgA classes could be specifically targeted using IgAimG or IgAimA. The exceptional efficacy and specificity of IgAim may make them superior to currently available therapeutic options for B-cell tumors.

Antibodies do not always defend against infections; in some instances, antibodies can even facilitate infections. The phenomena of antibody-dependent enhancement (ADE) [20] and original antigenic sin (OAS) [21] highlight the potential limitations and challenges associated with relying solely on antibodies for protection against infections. ADE occurs when the binding of a virus to suboptimal antibodies enhances viral entry into host cells and promotes replication [20]. On the other hand, OAS indicates the immune system’s tendency to cling to the memory based on a previous infection, which can limit its ability to respond to mutant strains effectively [21]. Applying IgAim to ADE or OAS could potentially help erase these negative immune memories and address these challenges.

Any of the ∼20 000 human genes can potentially give rise to an autoantigen, and the protein may become inactivated or activated depending on the antibody binding. While currently, the number of human diseases categorized as autoimmune diseases is limited [22], theoretically, there can be far >100 types of autoimmune diseases. By fusing the antigen with diphtheria toxin, specific personalized drugs for each autoimmune disease or allergy could be developed (Fig. 4). In cases where the protein has a considerable molecular weight, it may not be necessary to utilize the entire antigen; explicitly targeting the epitope by IgAim could be sufficient. This method can potentially eliminate antigen-specific memory by targeting the immune response.

Since IgAim was derived from SpA (Fig. 1D), IgAim is compatible with SpA variants, namely affibodies (Fig. 3). This compatibility opens up possibilities for more than just targeting antibodies. Like IgAimA, by fusing diphtheria toxin with an affibody, there are opportunities to expand the scope of targeted therapeutics. Affibodies with diverse binding specificities have already been developed [23], and new affibodies can be screened based on their specific binding [15]. This approach allows for a broader range of targets beyond B cells, enabling the application of targeted therapeutics to various medical contexts.

The human immune system is exceptional in its ability to remember a pathogen, implying that it does not easily forget. IgAim may serve as a valuable tool in the future to help erase harmful immune memory by precisely targeting and eliminating antigen-specific B cells. IgAim offers the potential to mitigate the negative consequences associated with autoimmune diseases, specifically eliminate malignant B cells, and potentially provide a therapeutic strategy, for instance, for ADE. Further research to apply IgAim *in vivo* will be a critical next step for validating this new therapeutic strategy.

## Supplementary Material

ugaf016_Supplemental_File

## Data Availability

The author confirms that the data supporting the findings of this study are available within the article and its supplementary materials.
