# Supplementary material for: IgAim: cell surface Ig-aimed immune memory erasers for the therapy of autoimmune diseases and B leukemia
Source: NAR Mol Med. 2025 Apr 30;2(2):ugaf016. doi: 10.1093/narmme/ugaf016 (PMC12429969; doi:10.1093/narmme/ugaf016)
Supplement: ugaf016_Supplemental_File [file ugaf016_Supplemental_File.pdf]

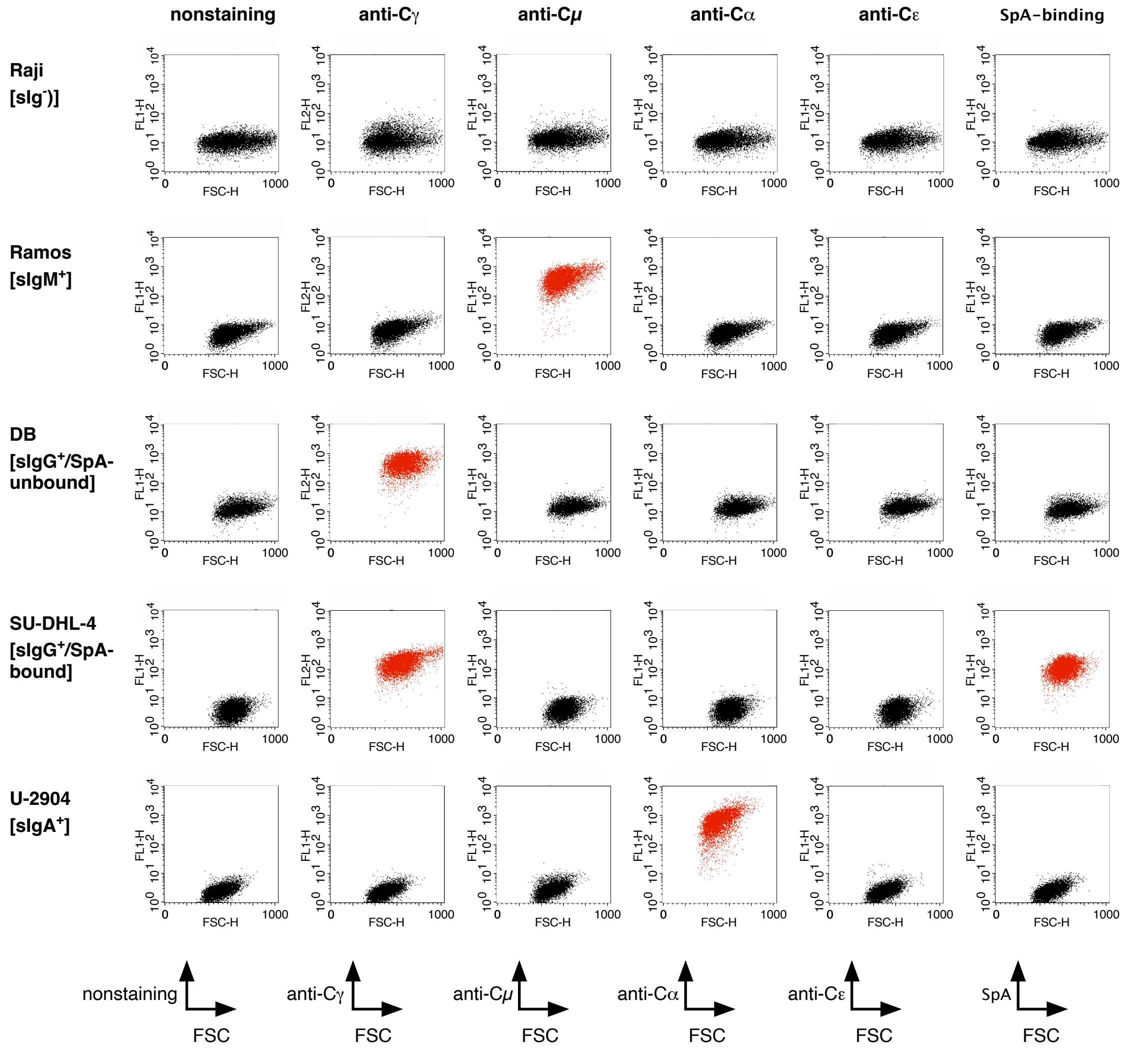

Supplementary Figure 1. Evaluation of B cell classes by flow cytometry. B cell classes were evaluated by antibody staining and SpA binding. The 30 B cells were classified into five categories: sIg^-, $sIgM^+$, $sIgG^+$/SpA-unbound, $sIgG^+$/SpA-bound, and $sIgA^+$. Of the 30 B cell lines analyzed, FACS profiles of representative cell lines in the five categories are shown. FACS profiles showing binding to the respective antibodies or SpA are highlighted in red.

A

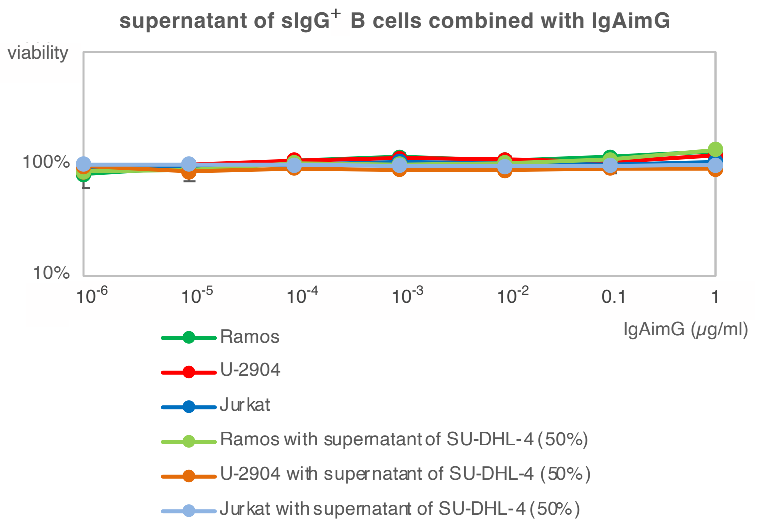

B

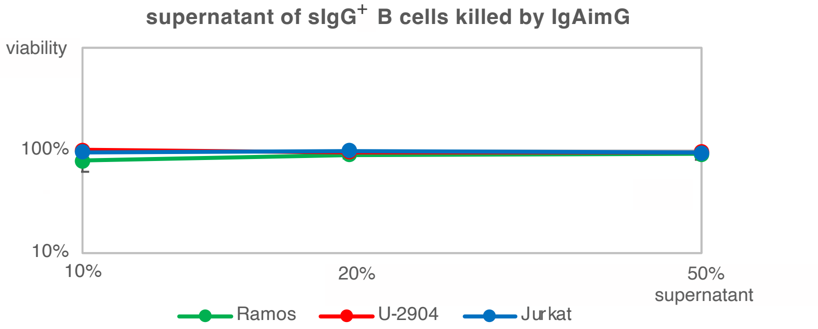

Supplementary Figure 2. Nonspecific cytotoxicity test of sIgG^{+} cell culture supernatants combined with IgAimG. (**A**) To figure out whether the cytotoxicity of IgAimG to sIgG^{+} B cells is mediated by cell surface IgG or cell-free IgG secreted from sIgG^{+} B cells, B cell lines were cultured in the medium containing 50% of the sIgG^{+} B cell line SU-DHL-4 culture supernatant. IgAimG in the presence of SU-DHL-4 culture supernatant was not cytotoxic to the Ramos B cell line (sIgM^{+}), U-2904 B cell line (sIgA^{+}), or Jurkat T cell line. This result indicates that IgAimG cytotoxicity to sIgG^{+} B cells is targeted to cell surface IgG rather than mediated by cell-free IgG. (**B**) The sIgG^{+} B cell line SU-DHL-4 was killed with 1 μg/ml of IgAimG for 3 days, and the supernatant was used to test whether sIgG^{+} B cells killed by IgAimG spread cytotoxic components around. The culture medium containing 50% of this supernatant was not cytotoxic to the Ramos B cell line (sIgM^{+}), the U-2904 B cell line (sIgA^{+}), or the Jurkat T cell line. This result indicates that cell contents released from IgAimG-killed cells are not cytotoxic to nonspecific cells. In these experiments, cells were cultured for 3 days under the conditions described above, and viability was measured by luminescent cell viability assay.
